# Supporting relationships, providing presence: Arts-informed approaches to LTC staff dementia care literacy

**DOI:** 10.1093/geroni/igaa057.3323

**Published:** 2020-12-16

**Authors:** Kelly O’Neil, Marco Redden, Katie Aubrecht

**Affiliations:** 1 St. Francis Xavier University, Antigonish, Nova Scotia, Canada; 2 Mount Saint Vincent University, Halifax, Nova Scotia, Canada

## Abstract

Early findings and insights are shared from an interpretive analysis of interviews with 15 leaders in arts-based approaches in dementia care. This was conducted as part of a larger project that has the goal of operationalizing ‘good’ literacy in social and relational care in long-term care (LTC). Interviews aimed to identify promising directions in arts-based approaches in education and training for LTC staff provincially, nationally, and internationally, and understand how they contribute to ‘good’ mental health and dementia literacy. Participants were recruited using a purposive snowball sampling method and semi-structured interview guide. Interviews were conducted in-person, via telephone and Zoom, and digitally recorded. Arts-making enhances mental health and dementia literacy of LTC staff by: 1) supporting relationships by generating trust and collaboration among persons living with mental health conditions and/or dementia, arts facilitators, and family members; 2) creating alternative communication spaces that allow people to see themselves and be seen by others from new perspectives; 3) fostering an artistic sensibility that: encourages imagination and empathy, brings spontaneity and playfulness to interactions, and disrupts restrictive expectations entrenched in typical caregiver/care receiver relationships. ‘Good’ literacy involves a relational and spatial awareness which manifests in the form of an artistic sensibility. Arts-based approaches can be used to enhance quality care by capacitating staff in the art of being open and curious, nimble and flexible, in how they know and make connections on an interpersonal level, in the moment.

